# Vaccine hesitancy among healthcare professionals: factors associated with continuity of the COVID-19 vaccination schedule

**DOI:** 10.1590/0034-7167-2025-0477

**Published:** 2026-07-31

**Authors:** Amanda Cristina Teixeira do Prado, Ana Laura Batista Silva, Gabriela Gonçalves Amaral, Selma Maria da Fonseca Viegas, Richardson Miranda Machado, Valéria Conceição de Oliveira, Eliete Albano de Azevedo Guimarães

**Affiliations:** I Universidade Federal de São João del-Rei. Divinópolis, Minas Gerais, Brazil; II Universidade de São Paulo. São Paulo, São Paulo, Brazil

**Keywords:** COVID-19, Immunization Schedule, Vaccination Hesitancy, Health Personnel, Health Evaluation., COVID-19, Esquemas de Inmunización, Vacilación a la Vacunación, Personal de Salud, Evaluación en Salud.

## Abstract

**Objectives:**

to analyze factors associated with healthcare professionals’ decisions regarding COVID-19 vaccination schedule continuation.

**Methods:**

an analytical cross-sectional study was conducted with 221 healthcare professionals working in public healthcare services in a regional hub municipality in Minas Gerais. A questionnaire was used with questions about sociodemographic factors, vaccination status, and the determinants of confidence, convenience, and compliance. The response variable was the number of doses of COVID-19 vaccines. Descriptive data analysis and logistic regression were performed.

**Results:**

it was identified that 21.5% of healthcare professionals hesitated to continue the COVID-19 vaccination schedule, with the sex (p=0.037; OR=2.163) and confidence in vaccine safety (p=0.003; OR=0.317) variables being associated with this vaccine hesitancy.

**Conclusions:**

there are professionals hesitant to continue being vaccinated against COVID-19. Interventions that strengthen confidence in vaccines and adopt continuous education strategies are essential.

## RESUMEN

**Objetivos:**

analizar los factores asociados a las decisiones de los profesionales sanitarios respecto a la continuación del calendario de vacunación contra la COVID-19.

**Métodos:**

se realizó un estudio transversal analítico con 221 profesionales sanitarios que trabajan en servicios de salud pública en un municipio con un centro regional en Minas Gerais. Se utilizó un cuestionario con preguntas sobre factores sociodemográficos, estado de vacunación y determinantes de confianza, conveniencia y cumplimiento. La variable de respuesta fue el número de dosis de vacunas contra la COVID-19. Se realizó un análisis descriptivo de datos y una regresión logística.

**Resultados:**

se identificó que el 21,5% de los profesionales sanitarios dudaban en continuar con el calendario de vacunación contra la COVID-19, y las variables sexo (p=0,037; OR=2,163) y confianza en la seguridad de la vacuna (p=0,003; OR=0,317) se asociaron con esta reticencia.

**Conclusiones:**

hay profesionales que dudan en continuar vacunándose contra la COVID-19. Las intervenciones que fortalezcan la confianza en las vacunas y adopten estrategias de educación continua son esenciales.

## INTRODUCTION

The emergence of the novel SARS-CoV-2 virus in Wuhan, China, which is highly transmissible and lethal, led the World Health Organization (WHO) to declare a pandemic and a Public Health Emergency of International Concern in 2020 due to its rapid spread^(1,2)^. As of August 18, 2025, Brazil had recorded 39,300,196 cases and 716,569 deaths^(3)^, ranking among the countries with the highest infection and mortality rates from COVID-19^(4)^. Healthcare professionals, especially nurses, were the most affected^(5)^.

Although measures such as the use of personal protective equipment and social isolation have been essential, vaccination has proven to be an effective strategy for controlling the pandemic and protecting healthcare professionals^(6)^. Since these professionals work directly with infected people, they were prioritized for vaccination^(7)^. In Brazil, initially, the CoronaVac, AstraZeneca, Pfizer and Janssen vaccines were used^(8)^, with a positive impact on reducing hospitalizations and deaths^(9,10)^.

As new variants emerged, the vaccines were updated. In 2023, a bivalent vaccine targeting the original strain and the Omicron variant was recommended^(11)^. In 2024, an updated vaccine was recommended in response to the XBB 1.5 variant, especially for healthcare professionals^(12,13)^. In 2025, the vaccination schedule began to include an annual booster dose with mRNA vaccines (Moderna, Pfizer, and Serum/Zalika)^(14)^.

However, the progress of vaccination was accompanied by misinformation and distrust regarding the safety and efficacy of vaccines, which affected adherence among healthcare professionals^(14,15)^. This group, particularly those in Primary Health Care, exerts a strong influence on the population’s acceptance of vaccines^(10,16,17)^.

Due to the reduced immune response to the initial vaccine regimen and the emergence of new variants, booster doses are recommended. However, hesitancy among healthcare professionals has been observed in relation to factors such as education level, income, sex, occupation, fear of adverse effects, and history of events supposedly attributable to vaccination (ESAVI). Vaccine hesitancy determinants include confidence, convenience, and compliance^(16,18-21)^.

Low participation compromises the effectiveness of COVID-19campaigns, vaccination coverage, and control^(21)^. Understanding this phenomenon is a global challenge. Therefore, it is essential to investigate the factors that influence the intention to continue vaccination among healthcare professionals^(18-20)^.

This group’s choice is justified by their greater exposure to the risk of infection and their central role in promoting vaccination^(22)^. Despite the clear benefits of vaccines, there is still hesitancy regarding booster doses^(23)^, which can impact immunization recommendations for the population. Therefore, it is important to identify the determinants of vaccine hesitancy among these professionals.

## OBJECTIVES

To analyze factors associated with healthcare professionals’ decisions regarding COVID-19 vaccination schedule continuation.

## METHODS

### Ethical aspects

This study is part of the research project “*Saúde, trabalho e segurança do profissional da saúde pública em tempos da pandemia de COVID-19*”, approved in 2022 by the *Universidade Federal de São João del-Rei* Research Ethics Committee, *Centro-Oeste Dona Lindu* campus.

### Study design, period and location

This is an analytical cross-sectional study guided by STrengthening the Reporting of OBservational studies in Epidemiology recommendations.

The study was conducted from November 2022 to April 2023 at the Occupational Health and Safety Reference Center (In Portuguese, *Centro de Referência em Saúde e Segurança do Trabalhador* - CRESST) in Divinópolis, a hub municipality in the Central-West macro-region of Minas Gerais, which encompasses 53 municipalities distributed across seven micro-regions. Divinópolis has an estimated population of 242,505 inhabitants, with a Municipal Human Development Index of 0.764^(24)^.

### Population and sample

The study population consisted of public healthcare service professionals (nurses, doctors, pharmacists, physiotherapists, dentists, nutritionists, psychologists, nursing technicians and assistants, laboratory technicians, radiology technicians, and community health workers) who were absent from work due to a confirmed case of SARS-CoV-2 infection and who reported their absence to CRESST between January 1, 2020, and July 31, 2022. During this period, 690 absences from work were recorded for healthcare professionals due to contracting COVID-19 (International Classification of Diseases B34.2).

For the sample size calculation, a proportion of 25.4% of professionals who were not vaccinated against influenza was considered^(22)^, a value that will provide the largest sample size for a finite population (n=690), setting the significance level at 5% (alpha or type I error), sampling error at 5%, and confidence level at 95%. Professionals who had worked for at least six months and were absent from work due to the virus were included in the study. Those who were not working during the data collection period, as well as those on leave, vacation, or any other type of absence, were excluded. Professionals who had worked for at least six months and were absent from work due to COVID-19 were included in the study. Those who were not working during the data collection period, as well as those on leave, vacation, or any other type of absence, were excluded.

### Study protocol

A total of 221 participants were selected via simple random sampling from CREEST’s electronic health monitoring system for workers. Initially, each selected professional received an individual invitation with a brief explanation of the research. Then, they were contacted by telephone to schedule a date and time for the interview. Data collection took place at CREEST after the Informed Consent Form was signed.

Data were collected using a self-administered structured questionnaire containing ten questions regarding participants’ sociodemographic factors and vaccination status, in addition to 12 questions about the determinants proposed by the 3Cs model: confidence, convenience, and compliance^(22,25)^.

Exposure variables included sociodemographic characteristics such as sex (female/male), education level (no education and incomplete elementary school, complete elementary school and incomplete high school, complete high school and incomplete higher education, complete higher education), race/color (white, brown, and black), individual income (more than one-quarter to half the minimum wage, half to one minimum wage, more than one to two minimum wages, more than two to three minimum wages, more than three to five minimum wages, more than five minimum wages), residence (urban, rural), and training (nursing professional, other). Moreover, the history of ESAVI after COVID-19 vaccination (yes, no) and variables related to the determinants predicted in the 3Cs model were analyzed^(22,25)^.

The 3Cs is an analysis model implemented by WHO^(25)^ and the Strategic Advisory Group of Experts on Immunization (SAGE) in 2012 to understand the concept of vaccine hesitancy and its determinants^(26)^. This model is composed of three determinants: confidence in the vaccine’s efficacy and safety, in healthcare services that offer the vaccines, and in healthcare professionals who are part of the vaccination process (confidence); population’s availability and accessibility to immunizations and healthcare services (convenience); and low perception of the importance of vaccination for controlling vaccine-preventable diseases (complacency)^(25,26)^.

In the confidence domain, the variables included experiences that led to distrust in vaccines (yes/no), confidence in vaccine efficacy (yes/no), confidence in information provided by healthcare professionals (yes/no), safety regarding the vaccine (yes/no), and confidence in healthcare services and professionals responsible for the vaccination process (yes/no). Regarding convenience, issues such as access to sufficient information to decide whether to vaccinate (yes/no), whether the individual felt informed about the risks/benefits of vaccination (yes/no), whether vaccination occurred due to a workplace campaign (yes/no), and whether vaccination was motivated by information about the benefits of vaccination in the media (yes/no) were considered. Finally, regarding compliance, attitudes such as the perception that working with many people increases the chances of contracting COVID-19 (agree/disagree), the perception that a person has much to gain by being vaccinated (agree/disagree), and the idea that, in order to be vaccinated, it is necessary to give up one’s own conceptions about the benefits, usefulness, and risks of vaccination (agree/disagree) were assessed^(22)^.

The response variable was the number of booster doses of the COVID-19 vaccine received (one dose, two doses, three doses, four doses). Healthcare professionals with four or more doses of the vaccine were considered to have adhered to vaccination schedule continuation, and professionals with up to three doses were considered reluctant to continue the vaccination schedule.

It is noteworthy that this stratification occurred within the primary vaccination schedule at the time of data collection, which included two vaccine doses. Healthcare professionals were advised to receive two additional booster doses if they began the primary schedule with CoronaVac, AstraZeneca, or Pfizer, and three booster doses if they began the primary schedule with Janssen^(8)^. In 2023, the bivalent vaccine was recommended for booster doses, and an annual booster dose was recommended for healthcare professionals, regardless of the number of doses they had received^(11)^.

### Data organization and analysis

Initially, a descriptive data analysis was performed. The age variable underwent the Shapiro-Wilk test and was found to be normal. Therefore, it is presented as median and quartiles (Q1 and Q3), and the comparison between those who decided to continue the COVID-19 vaccination schedule and those who did not was made using the Mann-Whitney test. For the comparison between hesitant and non-hesitant individuals regarding the other explanatory variables, the chi-square test was performed.

For the logistic regression model, all variables with a p-value less than 0.2 in the bivariate analyses were preselected. This criterion allows potentially relevant variables that may influence the final outcome to be included, even if they are not initially statistically significant. Next, we performed a progressive exclusion process, removing the least significant variables, i.e., those with a p-value exceeding 0.05, to ensure that only the variables significantly associated with the outcome remained in the final model.

All analyses were performed using IBM SPSS Statistics for Windows version 25.0, with a significance level of 5%.

## RESULTS

Of the 221 healthcare professionals, most were female (n=180; 81.4%) with a median age of 41 years (Q1=36 years; Q3=49 years). Most lived in urban areas (n=214; 97.7%), and 69.5% (n=153) earned up to two minimum wages. Additionally, 70.3% (n=152) had completed high school. Most participants worked in nursing (n=185; 84.1%). Furthermore, more than half of the professionals (n=148; 67.3%) reported experiencing at least one adverse event (Table 1).

**Table 1:** Factors associated with hesitancy in continuing the COVID-19 vaccination schedule according to socioeconomic variables, Divinópolis, Minas Gerais, Brazil, 2022-2023

Variables	Vaccine hesitancy			
	Total n (%)	No n (%)	Yes n (%)	*p* value^*^
Sex (n=221)				
Woman	180 (81.4)	134 (84.8)	46 (73.0)	**0.042**
Man	41 (19.1)	24 (15.2)	17 (27.0)	
Race/color (n=217)				
White	129 (59.4)	95 (61.3)	34 (54.8)	0.314
Brown	65 (30.0)	42 (27.1)	23 (37.1)	
Black	23 (10.6)	18 (11.6)	5 (8.1)	
Income, minimum wage† (n=220)				
More than a quarter to half the minimum wage	6 (2.7)	6 (3.8)	0 (0.0)	0.332
Half to one minimum wage	84 (38.2)	56 (35.4)	28 (45.2)	
One to two minimum wages	63 (28.6)	45 (28.5)	18 (29.0)	
Two to three minimum wages	31 (14.1)	23 (14.6)	8 (12.9)	
Three to five minimum wages	15 (6.8)	10 (6.3)	5 (8.1)	
More than five minimum wages	21 (9.5)	18 (11.4)	3 (4.8)	
Residence (n=219)				
Urban	214 (97.7)	155 (98.7)	59 (95.2)	0.139
Rural	5 (2.3)	2 (1.3)	3 (4.8)	
Education (n=216)				
No education and incomplete elementary school	2 (0.9)	2 (1.3)	0 (0.0)	0.738
Complete elementary school and incomplete high school	91 (42.1)	65 (41.7)	26 (43.3)	
Complete high school and incomplete higher education	59 (27.3)	41 (26.3)	18 (30.0)	
Complete higher education	64 (29.6)	48 (30.8)	16 (26.7)	
Training (n=220)				
Nursing professionals	185 (84.1)	133 (84.2)	52 (83.9)	0.955
Other	35 (15.9)	25 (15.8)	10 (16.1)	
Had an event supposedly attributable to vaccination or immunization (n=220)				
No	72 (32.7)	52 (33.1)	20 (31.7)	0.844
Yes	148 (67.3)	105 (66.9)	43 (68.3)	

* Chi-square test;

† Reference value for the minimum wage in Brazil in 2022=R$1.212.00.

Among participants, 71.5% (n=158) received four or more doses of the vaccine, indicating that they were willing to complete the vaccination schedule, and 21.5% (n=63) received up to three doses of the vaccine, therefore being hesitant to continue the COVID-19 vaccination schedule.

As shown in Table 1, bivariate analysis revealed that only sex was associated with vaccine hesitancy regarding COVID-19 vaccination schedule continuation (p=0.042).

This study did not reveal any significant differences between the two groups with respect to age (p=0.408). However, when analyzing the factors associated with the decision to continue the COVID-19 vaccination schedule, a statistically significant association was observed in the variables (C1, C4, and L3) and components of the determinants of confidence, compliance and convenience of vaccine hesitancy (Table 2).

**Table 2:** Factors associated with hesitancy in continuing the COVID-19 vaccination schedule according to the confidence, complacency, and convenience determinants, Divinópolis, Minas Gerais, Brazil, 2022-2023

Variables	Vaccine hesitancy			
	Total n (%)	No n (%)	Yes n (%)	*p* value^^*^^
Confidence (C)				
Experience - vaccines (C1): Has anything ever happened in your life or community that made you stop believing in vaccines? (n=215)				
No	193 (89.8)	141 (92.8)	52 (82.5)	**0.024**
Yes	22 (10.2)	11 (7.2)	11 (17.5)	
Government - vaccine (C2): Do you believe the government provides the best vaccine on the market? (n=213)				
No	66 (31.0)	42 (27.8)	24 (38.7)	0.118
Yes	147 (69.0)	109 (72.2)	38 (61.3)	
Confidence - information (C3): Do you trust the information that professionals give you about vaccination? (n=216)				
No	12 (5.6)	6 (3.9)	6 (9.5)	0.102
Yes	204 (94.4)	147 (96.1)	57 (90.5)	
Safety - vaccine (C4): Do you feel safe receiving new vaccines? (n=216)				
No	34 (15.7)	17 (11.1)	17 (27.0)	**0.004**
Yes	182 (84.3)	136 (88.9)	46 (73.0)	
Confidence - professionals (C5): Do you trust the people who manage the vaccination process at the Family Health Unit? (n=216)				
No	8 (3.7)	6 (3.9)	2 (3.2)	0.999
Yes	208 (96.3)	147 (96.1)	61 (96.8)	
**Convenience (V)**				
Knowledge - Vaccination campaigns (V1): In vaccination campaigns, do you have enough information to decide whether to be vaccinated? (n=216)				
No	25 (11.6)	14 (9.2)	11 (17.5)	0.083
Yes	191 (88.4)	139 (90.8)	52 (82.5)	
Knowledge - Risks/benefits (V2): Do you feel sufficiently informed about the risks/benefits of vaccination? (n=215)				
No	48 (22.3)	35 (23.0)	13 (20.6)	0.702
Yes	167 (77.7)	117 (77.0)	50 (79.4)	
Campaigns - Workplace vaccination (V3): I was vaccinated against COVID-19 because a vaccination campaign was held at my workplace (n=216)				
No	89 (41.2)	67 (43.8)	22 (34.9)	0.229
Yes	127 (58.8)	86 (56.2)	41 (65.1)	
Information - social media (V4): I was vaccinated against COVID-19 after hearing information about the benefits of the vaccine in the media (television, radio, social media) (n=216)				
No	44 (20.4)	28 (18.3)	16 (25.4)	0.239
Yes	172 (79.6)	125 (81.7)	47 (74.6)	
**Compliance (L)**				
Work - transmission (L1): Working with many people every day increases my chances of catching COVID-19 (n=216)				
Disagree	23 (10.6)	14 (9.2)	9 (14.3)	0.266
Agree	193 (89.4)	139 (90.8)	54 (85.7)	
Benefits - vaccination (L2): I have much to gain by being vaccinated against COVID-19 (n=216)				
Disagree	5 (2.3)	2 (1.3)	3 (4.8)	0.15
Agree	211 (97.7)	151 (98.7)	60 (95.2)	
Conceptions - vaccination (L3): To be vaccinated against COVID-19, I would need to give up my conceptions about the usefulness, benefits, and risks of vaccination (n=216)				
Disagree	170 (78.7)	126 (82.4)	44 (69.8)	**0.041**
Agree	46 (21.3)	27 (17.6)	19 (30.2)	

* *Chi-square test.*

Table 3 shows the results of the logistic regression analysis of factors associated with vaccine hesitancy regarding the completion of the recommended schedule for SARS-CoV-2 vaccine administration among healthcare professionals. Male participants are 2.163 times more likely to exhibit vaccine hesitancy than female participants. Additionally, professionals who reported feeling safe receiving new vaccines were 68.3% less likely to exhibit vaccine hesitancy than those who did not feel safe. The sex and safety regarding vaccines variables explain 72.2% of the variability in responses to vaccine hesitancy.

**Table 3:** Results of logistic regression for factors associated with vaccine hesitancy among healthcare professionals, Divinópolis, Minas Gerais, Brazil, 2022-2023

Variables	Vaccine hesitancy			
	Odds Ratio	Wald test	*p* value^*^	95% CI
Sex	2.163	4.357	0.037	1.048 - 4.465
Safety - vaccine (C4)	0.317	8.712	0.003	0.148 - 0.680
Constant	0.896	0.099	0.753	

* *Chi-square test; CI - Confidence interval; response variable: vaccine hesitancy. Reference categories: sex (female), vaccine safety (no); classification table: 72.2%.*

## DISCUSSION

The rapid development of COVID-19 vaccines and the urgency of their application to contain the pandemic have raised questions about safety and efficacy, contributing to low vaccination rates^(9)^. Despite the proven benefits, some healthcare professionals still hesitate to adhere to the vaccination schedule. This compromises individual protection, disease control, and the achievement of coverage goals^(27)^. This study found that hesitancy was primarily associated with sex and confidence in vaccine safety. These results highlight the influence of sociodemographic and perceptual factors on the decision to continue immunization.

Among the analyzed variables, males exhibited a higher likelihood of hesitating to continue the recommended schedule for the administration of the SARS-CoV-2 vaccine compared to females. Similar findings were reported in Brazil, where a study of 173,178 participants identified a significant association between male sex and vaccine hesitancy (OR=1.62)^(28)^, and in Venezuela, where a higher proportion of men were unvaccinated compared to vaccinated men (p=0.037)^(29)^. Research in Poland indicates that women showed a greater willingness to receive booster doses, attributed to a higher perception of the importance of preventive measures^(30)^ and concern about the consequences of COVID-19^(31)^. In contrast, men showed less preventive engagement, influenced by attitudes of masculinity and a sense of invulnerability^(32,33)^, which may have contributed to higher infection rates among men during the pandemic^(34)^.

Despite this, not all contexts present the same pattern. Studies carried out in Thailand^(35)^ and Austria^(36)^ observed lower adherence among women, attributed to the belief that vaccination could cause infertility^(37)^. Furthermore, research with healthcare professionals in 23 countries found no association between sex and vaccine hesitancy^(38)^, showing that this factor can vary according to cultural, social and informational aspects.

Vaccine hesitancy among healthcare professionals is especially concerning. In addition to compromising coverage among these workers, it directly influences public acceptance. This is because a hesitant professional tends not to actively recommend vaccination^(36,39)^. This behavior increases the risk of infection for healthcare professionals and patients, as many serious cases and deaths occur in unvaccinated individuals^(40)^. A study assessing the immunogenicity of AstraZeneca after prolonged intervals between doses demonstrated that continuing the vaccination schedule with booster doses is necessary to maintain high levels of immunity^(41)^.

The need for booster shots is reinforced by the high occupational exposure of healthcare professionals and the constant emergence of variants, both of which contribute to declining immunity and reduced vaccine efficacy^(42)^. Reviews indicate a significant drop in protection about six months after the primary regimen^(42)^, justifying the recommendation of annual boosters, especially for this high-risk group^(43)^. Clinical trials, such as one assessing BNT162b2 (Pfizer-BioNTech), confirm a significant increase in immune response after an additional dose^(44)^.

Nevertheless, even in the face of robust evidence on the benefit of reinforcement^(45)^, some professionals remain hesitant, with nurses showing a greater delay compared to other categories^(46)^. Confidence in vaccine safety was one of the most relevant predictors among the classic determinants of hesitancy (confidence, convenience, and complacency), supporting findings from India^(47,48)^ and multinational research with professionals from 22 countries^(49)^. Lack of confidence can stem from doubts about efficacy, fear of adverse events, and distrust in pharmaceutical authorities or industries^(36,50)^.

Although there was no statistically significant association, more than half of the participants reported experiencing ESAVI. Research in different countries suggests that fear of ESAVI is a factor that potentiates hesitancy, as observed in studies in India^(47)^, Asia and Africa^(50)^, and in other international investigations^(48,51)^.

### Study limitations

A limitation of this study is its cross-sectional design, which only allows for the identification of associations between the analyzed factors and vaccine hesitancy, not the establishment of causal relationships. In addition, the use of a self-administered questionnaire may have introduced information bias, either due to inaccurate recall (memory bias) or participants’ tendency to provide socially desirable answers.

### Contributions to nursing, health, or public policy

This study addresses vaccine hesitancy among healthcare professionals regarding the continuation of the COVID-19 vaccination program. This behavior threatens the safety of these workers and patients, reinforcing the need for actions that encourage adherence to the complete vaccination schedule. For nursing practice, the findings underscore the importance of ongoing educational strategies that empower professionals to disseminate reliable, evidence-based information. In public health, the findings contribute to the planning of targeted interventions for specific groups by considering sociodemographic and perceptual factors. Regarding public policy, the findings underscore the necessity of clear, transparent, and segmented communication to combat misinformation, bolster confidence in vaccines, and sustain high vaccination rates.

## CONCLUSIONS

An analysis of factors associated with hesitancy in completing the recommended schedule of doses of the SARS-CoV-2 vaccine revealed that confidence in vaccine safety is crucial for adherence among healthcare professionals. Gender was also found to be a relevant factor in shaping attitudes toward immunization. Some of these professionals, particularly nursing staff, showed resistance to continuing vaccination, which reinforces the need for targeted strategies for this group.

The findings of this study can inform interventions that promote adherence to the continuity of the COVID-19 vaccination schedule. These interventions should emphasize demystifying misconceptions and misinformation about vaccines, promoting transparency regarding their efficacy and safety, and reinforcing their benefits in controlling vaccine-preventable diseases.

Additionally, it is important to conduct research that deepens our understanding of the factors that influence vaccination decisions among healthcare professionals. This will enable us to implement effective healthcare service strategies that increase acceptance and ensure the protection of this group and the population they serve.

## Data Availability

The research data are available within the article.
