# Supplementary material for: Vaccine hesitancy among healthcare professionals: factors associated with continuity of the COVID-19 vaccination schedule
Source: Rev Bras Enferm. 2026 Jul 31;79:e20250477. doi: 10.1590/0034-7167-2025-0477 (PMC13435866; doi:10.1590/0034-7167-2025-0477)
Supplement: Translation

# Translation: Hesitação vacinal entre profissionais de saúde: fatores associados à continuidade do esquema contra a COVID-19

**DOI:** 10.1590/0034-7167-2025-0477pt

**Authors:** Amanda Cristina Teixeira do Prado, Ana Laura Batista Silva, Gabriela Gonçalves Amaral, Selma Maria da Fonseca Viegas, Richardson Miranda Machado, Valéria Conceição de Oliveira, Eliete Albano de Azevedo Guimarães

**Affiliations:** I Universidade Federal de São João del-Rei. Divinópolis, Minas Gerais, Brasil; II Universidade de São Paulo. São Paulo, São Paulo, Brasil

**Keywords:** COVID-19, Esquemas de Imunização, Hesitação Vacinal, Pessoal de Saúde, Avaliação em Saúde.

## Abstract

**Objetivos:**

analisar os fatores associados na decisão dos profissionais de saúde sobre a continuidade do esquema vacinal contra a COVID-19.

**Métodos:**

estudo transversal analítico, realizado com 221 profissionais de saúde que atuavam nos serviços públicos de saúde de um município polo regional de Minas Gerais. Utilizou-se um questionário com questões sobre fatores sociodemográficos, condições de vacinação, e determinantes confiança, conveniência e complacência. A variável resposta foi o número de doses das vacinas contra a COVID-19. Foram realizadas análise descritiva dos dados e regressão logística.

**Resultados:**

identificou-se que 21,5% dos profissionais de saúde hesitaram em continuar o esquema vacinal contra a COVID-19, sendo as variáveis sexo (p=0,037; OR=2,163) e confiança na segurança das vacinas (p=0,003; OR=0,317) associadas a essa hesitação vacinal.

**Conclusões:**

há profissionais hesitantes em continuar a se vacinar contra a COVID-19. Intervenções que fortaleçam a confiança nas vacinas e adotem estratégias contínuas de educação são essenciais.

## INTRODUÇÃO

O surgimento do novo coronavírus (SARS-CoV-2), identificado em Wuhan (China), de alta transmissibilidade e letalidade, levou à declaração de pandemia em 2020 e de Emergência de Saúde Pública de Preocupação Internacional pela Organização Mundial da Saúde (OMS), devido à rápida disseminação do vírus^(1,2)^. Até 18 de agosto de 2025, o Brasil registrou 39.300.196 casos e 716.569 óbitos^(3)^, figurando entre os países com maiores taxas de infecção e mortalidade pela COVID-19^(4)^. Profissionais de saúde, especialmente de enfermagem, foram os mais afetados^(5)^.

Embora medidas como uso de equipamentos de proteção individual e isolamento social tenham sido essenciais, a vacinação se destacou como estratégia eficaz no controle da pandemia e na proteção dos profissionais de saúde^(6)^. Por atuar diretamente no cuidado a pessoas infectadas, esses profissionais foram priorizados para vacinação^(7)^. No Brasil, inicialmente, foram utilizadas as vacinas CoronaVac, AstraZeneca, Pfizer e Janssen^(8)^, com impacto positivo na redução de hospitalizações e óbitos^(9,10)^.

Com o surgimento de novas variantes, houve atualização dos imunizantes. Em 2023, recomendou-se a vacina bivalente para a cepa original e a variante Ômicron^(11)^. Em 2024, diante da variante XBB 1.5, indicou-se vacina atualizada, especialmente para profissionais de saúde^(12,13)^. Em 2025, o esquema vacinal passou a prever dose de reforço anual com vacinas RNAm (Moderna, Pfizer e Serum/Zalika)^(14)^.

No entanto, o avanço da vacinação foi acompanhado por desinformação e desconfiança sobre a segurança e eficácia dos imunizantes, afetando a adesão, inclusive, entre profissionais de saúde^(14,15)^. Esse grupo, especialmente na Atenção Primária à Saúde, exerce forte influência sobre a população na aceitação vacinal^(10,16,17)^.

Diante da redução da resposta imune ao esquema primário e do surgimento de novas variantes, foram recomendadas doses de reforço. Porém, observa-se hesitação entre profissionais de saúde relacionada a fatores como escolaridade, renda, sexo, ocupação, medo de efeitos adversos, histórico de eventos supostamente atribuíveis à vacinação (ESAVI) e os determinantes da hesitação vacinal confiança, conveniência e complacência^(16,18-21)^.

A baixa adesão compromete a eficácia das campanhas, a cobertura vacinal e o controle da COVID-19^(21)^. Compreender esse fenômeno é um desafio global. Assim, torna-se essencial investigar os fatores que influenciam a intenção de continuidade da vacinação entre profissionais de saúde^(18-20)^.

A escolha por esse grupo se justifica pela sua maior exposição ao risco de infecção e papel central na promoção da vacinação^(22)^. Apesar dos benefícios evidentes das vacinas, ainda há hesitação quanto às doses de reforço^(23)^, o que pode afetar a recomendação de imunização à população. Destaca-se, portanto, a importância de identificar os determinantes da hesitação vacinal entre esses profissionais.

## OBJETIVOS

Analisar os fatores associados à decisão dos profissionais de saúde sobre a continuidade do esquema vacinal contra COVID-19.

## MÉTODOS

### Aspectos éticos

Este estudo é parte da pesquisa “Saúde, trabalho e segurança do profissional da saúde pública em tempos da pandemia de COVID-19”, aprovada em 2022 pelo Comitê de Ética em Pesquisa da Universidade Federal de São João del-Rei, *campus* Centro-Oeste Dona Lindu.

### Desenho, período e local do estudo

Trata-se de estudo transversal analítico norteado pelas recomendações do *STrengthening the Reporting of OBservational studies in Epidemiology*.

Foi realizado no período de novembro de 2022 a abril de 2023 no Centro de Referência em Saúde e Segurança do Trabalhador (CRESST) de Divinópolis, um município polo da macrorregião Centro-Oeste de Minas Gerais que engloba 53 municípios distribuídos em sete microrregiões. Divinópolis possui uma população estimada em 242.505 habitantes, com um Índice de Desenvolvimento Humano Municipal de 0,764^(24)^.

### População e amostra

A população elegível para o estudo foi composta pelos profissionais que atuavam nos serviços públicos de saúde (enfermeiros, médicos, farmacêuticos, fisioterapeutas, odontólogos, nutricionistas, psicólogos, técnicos e auxiliares de enfermagem, técnicos de laboratório, técnicos de radiologia, e agentes comunitários de saúde), que tiveram licença do trabalho em detrimento do acometimento pela COVID-19 e notificados no CRESST entre 01 de janeiro de 2020 e 31 de julho de 2022. Foram registradas, no período, 690 licenças do trabalho de profissionais de saúde, em decorrência do acometimento por COVID-19 (Classificação Internacional das Doenças B34.2).

Para o cálculo amostral, foi considerada uma proporção de 25,4% de profissionais que não se vacinaram contra a influenza^(22)^, valor este que fornecerá o maior tamanho amostral para população finita (n=690), fixando o nível de significância em 5% (alfa ou erro tipo I), erro amostral, em 5%, e nível de confiança, em 95%. O número amostral estimado foi de 221 profissionais de saúde. Foram incluídos os profissionais que atuaram por um período mínimo de seis meses e tiveram afastamento do trabalho em decorrência do acometimento por COVID-19. Foram excluídos aqueles que não se encontravam em exercício profissional no período da coleta de dados e os que estavam em licença, férias, folga ou qualquer outro tipo de afastamento.

### Protocolo do estudo

Os 221 participantes foram escolhidos de forma aleatória simples no sistema eletrônico de acompanhamento da saúde dos trabalhadores do CREEST. Inicialmente, foi enviado um convite individual para os profissionais selecionados com a breve explicação sobre a pesquisa. A seguir, realizou-se contato telefônico para agendamento de data e horário da entrevista. A coleta de dados foi realizada no CREEST, após a assinatura do Termo de Consentimento Livre e Esclarecido.

Os dados foram coletados por meio de questionário estruturado autoaplicável contendo dez questões referentes aos fatores sociodemográficos e condições de vacinação do participante, além de 12 questões sobre os determinantes confiança, conveniência e complacência propostos pelo modelo 3Cs: confiança, conveniência e complacência^(22,25)^.

As variáveis de exposição incluíram características sociodemográficas, como sexo (feminino/masculino), escolaridade (sem instrução e com ensino fundamental incompleto, fundamental completo e ensino médio incompleto, médio completo e superior incompleto, superior completo), raça/cor (branca, parda e preta), renda individual (mais de um quarto a meio salário mínimo, de meio a um salário mínimo, mais de um a dois salários mínimos, mais de dois a três salários mínimos, mais de três a cinco salários mínimos, mais de cinco salários mínimos), residência (urbana, rural), formação (profissional da enfermagem, outro). Além disso, foi analisada a história de ESAVI após a aplicação da vacina contra a COVID-19 (sim, não) e as variáveis referentes aos determinantes previstos no modelo 3Cs^(22,25)^.

O 3Cs é um modelo de análise implementado pela OMS^(25)^ e pelo Grupo Consultivo Estratégico de Especialistas em Imunização (SAGE - *Strategic Adivisory Group of Experts on Immunization*), no ano de 2012, para a compreensão do conceito hesitação vacinal e seus determinantes^(26)^. Esse modelo é composto por três determinantes, sendo confiança na eficácia e segurança da vacina, nos serviços de saúde que ofertam as vacinas, e nos profissionais de saúde que fazem parte do processo de vacinação (confiança); disponibilidade e acessibilidade da população aos imunizantes e aos serviços de saúde (conveniência); e baixa percepção sobre a importância da vacinação para controle de doenças imunopreveníveis (complacência)^(25,26)^.

No domínio da confiança, as variáveis incluíram experiências que levaram a desacreditar das vacinas (sim/não), confiança na eficácia da vacina (sim/não), confiança nas informações fornecidas por profissionais de saúde (sim/não), segurança em relação à vacina (sim/não), e confiança nos serviços de saúde e nos profissionais responsáveis pelo processo de vacinação (sim/não). Em relação à conveniência, foram consideradas questões como acesso a informações suficientes para decidir se vacinar (sim/não), se o indivíduo se sentiu informado sobre os riscos/benefícios da vacinação (sim/não), se a vacinação ocorreu devido à campanha no local de trabalho (sim/não) e se a vacinação foi motivada por informações sobre os benefícios da vacinação nos meios de comunicação (sim/não). Por fim, no aspecto da complacência, foram avaliadas atitudes como a percepção de que trabalhar com muitas pessoas aumenta as chances de contrair COVID-19 (concordo/discordo), a percepção de que a pessoa tem muito a ganhar ao se vacinar (concordo/discordo) e a ideia de que, para se vacinar, é necessário abrir mão das próprias concepções sobre os benefícios, utilidade e riscos da vacinação (concordo/discordo)^(22)^.

A variável de resposta foi o quantitativo de doses de reforço recebidas da vacina contra COVID-19 (uma dose, duas doses, três doses, quatro doses). Profissionais de saúde com quatro ou mais doses da vacina foram considerados como tendo aderido à continuidade do esquema vacinal, e os profissionais com até três doses da vacina foram considerados relutantes à continuidade do esquema vacinal.

Destaca-se que esta estratificação se deu no esquema primário de vacinação, no momento da coleta de dados, que incluía duas doses de vacina, sendo indicado para o profissional de saúde o recebimento de mais duas doses de reforço para aqueles que iniciaram o esquema primário com CoronaVac, AstraZeneca e Pfizer, e três doses de reforço para quem iniciou esquema primário com Janssen^(8)^. No ano de 2023, foi recomendado o uso da vacina bivalente para aplicação de doses de reforço, e foi indicada uma dose de reforço anual para os profissionais de saúde, independentemente da quantidade de doses recebidas^(11)^.

### Organização e análise dos dados

Inicialmente, foi realizada a análise descritiva dos dados. A variável idade passou pelo tese de Shapiro-Wilk e teve a normalidade rejeitada. Em virtude disso, está apresentada em forma de mediana e quartis (Q1 e Q3), e a comparação entre aqueles que decidiram continuar o esquema vacinal da COVID-19 e aqueles que não deram continuidade foi feita através do teste de Mann-Whitney. Para a comparação entre hesitantes e não hesitantes em relação às demais variáveis explicativas, foi realizado o teste qui-quadrado.

Para a regressão logística, todas as variáveis que apresentaram valor de p inferior a 0,2 nas análises bivariadas foram pré-selecionadas para o modelo. Esse critério permite incluir variáveis potencialmente relevantes que podem influenciar o desfecho final, mesmo que inicialmente não sejam estatisticamente significativas. A seguir, um processo de exclusão progressiva foi realizado, removendo as variáveis menos significativas, ou seja, aquelas cujo valor de p excedia 0,05, para garantir que apenas as variáveis significativamente associadas ao desfecho permanecessem no modelo final.

Todas as análises foram feitas no IBM SPSS *Statistics for Windows* versão 25.0, com nível de significância de 5%.

## RESULTADOS

Entre os 221 profissionais de saúde, a maioria era do sexo feminino (n=180; 81,4%), com idade mediana de 41 anos (Q1= 36 anos; Q3=49 anos). A maior parte deles residia na zona urbana (n=214; 97,7%); 69,5% (n=153) tinham renda de até dois salários mínimos; e 70,3% (n=152) tinham ensino médio completo. A maioria dos participantes atuava na área da enfermagem (n=185; 84,1%). Além disso, mais da metade dos profissionais (n=148; 67,3%) relatou a ocorrência de algum ESAVI (Tabela 1).

**Tabela 1:** Fatores associados à hesitação na continuidade do esquema vacinal contra COVID-19 segundo variáveis socioeconômicas, Divinópolis, Minas Gerais, Brasil, 2022-2023

Variáveis	Hesitação vacinal			
	Total n (%)	Não n (%)	Sim n (%)	Valor de p^*^
Sexo (n = 221)				
Mulher	180 (81,4)	134 (84,8)	46 (73,0)	**0,042**
Homem	41 (19,1)	24 (15,2)	17 (27,0)	
Cor/raça (n = 217)				
Branca	129 (59,4)	95 (61,3)	34 (54,8)	0,314
Parda	65 (30,0)	42 (27,1)	23 (37,1)	
Preta	23 (10,6)	18 (11,6)	5 (8,1)	
Renda, salário mínimo^†^ (n = 220)				
Mais de um quarto a meio salário mínimo	6 (2,7)	6 (3,8)	0 (0,0)	0,332
De meio a um salário mínimo	84 (38,2)	56 (35,4)	28 (45,2)	
De um a dois salários mínimos	63 (28,6)	45 (28,5)	18 (29,0)	
De dois a três salários mínimos	31 (14,1)	23 (14,6)	8 (12,9)	
De três a cinco salários mínimos	15 (6,8)	10 (6,3)	5 (8,1)	
Mais de cinco salários mínimos	21 (9,5)	18 (11,4)	3 (4,8)	
Residência (n = 219)				
Urbana	214 (97,7)	155 (98,7)	59 (95,2)	0,139
Rural	5 (2,3)	2 (1,3)	3 (4,8)	
Escolaridade (n = 216)				
Sem instrução e com ensino fundamental incompleto	2 (0,9)	2 (1,3)	0 (0,0)	0,738
Fundamental completo e ensino médio incompleto	91 (42,1)	65 (41,7)	26 (43,3)	
Médio completo e superior incompleto	59 (27,3)	41 (26,3)	18 (30,0)	
Superior completo	64 (29,6)	48 (30,8)	16 (26,7)	
Formação (n = 220)				
Profissionais de enfermagem	185 (84,1)	133 (84,2)	52 (83,9)	0,955
Outro	35 (15,9)	25 (15,8)	10 (16,1)	
Teve evento supostamente atribuível à vacinação ou imunização (n = 220)				
Não	72 (32,7)	52 (33,1)	20 (31,7)	0,844
Sim	148 (67,3)	105 (66,9)	43 (68,3)	

* Teste qui-quadrado;

† Valor de referência do salário mínimo no Brasil em 2022 = R$1.212,00.

Entre os participantes, 71,5% (n=158) tomaram quatro e mais doses da vacina contra a COVID-19, ou seja, não hesitantes à continuidade do esquema vacinal, e 21,5% (n=63) tomaram até três doses da vacina, sendo, portanto, hesitantes à continuidade do esquema vacinal contra COVID-19.

Na análise bivariada apresentada na Tabela 1, apenas a variável sexo esteve associada à hesitação vacinal na continuidade do esquema vacinal contra COVID-19 (p=0,042).

Este estudo não evidenciou diferenças entre os dois grupos em relação a idade (*p* = 0,408). Ao analisar os fatores associados na decisão de continuar o esquema da COVID-19, observou-se associação estatisticamente significativa nas variáveis (C1, C4 e L3) e componentes dos determinantes confiança, complacência e conveniência da hesitação vacinal (Tabela 2).

**Tabela 2:** Fatores associados à hesitação na continuidade do esquema vacinal contra COVID-19 segundo os determinantes confiança, complacência e conveniência, Divinópolis, Minas Gerais, Brasil, 2022-2023

Variáveis	Hesitação vacinal			
	Total n (%)	Não n (%)	Sim n (%)	Valor de p^^*^^
Confiança (C)				
Experiência - vacinas (C1): Já aconteceu algo na sua vida ou comunidade que fez com que você deixasse de acreditar nas vacinas? (n = 215)				
Não	193 (89,8)	141 (92,8)	52 (82,5)	**0,024**
Sim	22 (10,2)	11 (7,2)	11 (17,5)	
Governo - vacina (C2): Você acredita que o governo fornece a melhora vacina do mercado? (n = 213)				
Não	66 (31,0)	42 (27,8)	24 (38,7)	0,118
Sim	147 (69,0)	109 (72,2)	38 (61,3)	
Confiança - informações (C3): Você confia em informações que os profissionais lhe repassam sobre a vacinação? (n = 216)				
Não	12 (5,6)	6 (3,9)	6 (9,5)	0,102
Sim	204 (94,4)	147 (96,1)	57 (90,5)	
Segurança - vacina (C4): Você se sente seguro para receber novas vacinas? (n = 216)				
Não	34 (15,7)	17 (11,1)	17 (27,0)	**0,004**
Sim	182 (84,3)	136 (88,9)	46 (73,0)	
Confiança - profissionais (C5): Você confia nas pessoas que cuidam do processo de vacinação da Unidade Saúde da Família? (n = 216)				
Não	8 (3,7)	6 (3,9)	2 (3,2)	0,999
Sim	208 (96,3)	147 (96,1)	61 (96,8)	
**Conveniência (V)**				
Conhecimento - campanhas vacinação (V1): Nas campanhas de vacinação, você tem informações suficientes para decidir se vacinar? (n = 216)				
Não	25 (11,6)	14 (9,2)	11 (17,5)	0,083
Sim	191 (88,4)	139 (90,8)	52 (82,5)	
Conhecimentos - riscos/benefícios (V2): Você se sente informado suficiente sobre riscos/benefícios da vacinação? (n = 215)				
Não	48 (22,3)	35 (23,0)	13 (20,6)	0,702
Sim	167 (77,7)	117 (77,0)	50 (79,4)	
Campanhas - vacinação trabalho (V3): Eu me vacinei contra COVID-19 porque foi realizada campanha de vacinação no meu trabalho (n = 216)				
Não	89 (41,2)	67 (43,8)	22 (34,9)	0,229
Sim	127 (58,8)	86 (56,2)	41 (65,1)	
Informações - mídias sociais (V4): Eu me vacinei contra COVID-19 após ouvir informações sobre os benefícios da vacina nos meios de comunicação (televisão, rádio, redes sociais) (n = 216)				
Não	44 (20,4)	28 (18,3)	16 (25,4)	0,239
Sim	172 (79,6)	125 (81,7)	47 (74,6)	
**Complacência (L)**				
Trabalho - transmissão (L1): Trabalhar com muitas pessoas todo dia aumentar minhas chances de pegar COVID-19 (n = 216)				
Discordo	23 (10,6)	14 (9,2)	9 (14,3)	0,266
Concordo	193 (89,4)	139 (90,8)	54 (85,7)	
Benefícios - vacinação (L2): Eu tenho muito a ganhar ao me vacinar contra COVID-19 (n = 216)				
Discordo	5 (2,3)	2 (1,3)	3 (4,8)	0,15
Concordo	211 (97,7)	151 (98,7)	60 (95,2)	
Concepções - vacinação (L3): Para me vacinar contra COVID-19, eu precisaria abrir mão das minhas concepções sobre utilidade, benefícios e riscos da vacinação (n = 216)				
Discordo	170 (78,7)	126 (82,4)	44 (69,8)	**0,041**
Concordo	46 (21,3)	27 (17,6)	19 (30,2)	

* *teste qui-quadrado.*

A Tabela 3 apresenta os resultados da regressão logística para fatores associados à hesitação vacinal na continuidade do esquema vacinal contra COVID-19 entre profissionais de saúde. Os participantes do sexo masculino têm 2,163 vezes mais chance de apresentar hesitação vacinal em comparação ao sexo feminino. Além disso, os profissionais que relataram se sentir seguros para receber novas vacinas têm 68,3% menos chance de apresentar hesitação vacinal em relação àqueles que não se sentem seguros. As variáveis sexo e segurança em relação às vacinas explicam 72,2% da variabilidade nas respostas de hesitação vacinal.

**Tabela 3:** Resultados da regressão logística para fatores associados à hesitação vacinal entre profissionais de saúde, Divinópolis, Minas Gerais, Brasil, 2022-2023

Variáveis	Hesitação vacinal			
	*Odds Ratio*	Teste de Wald	Valor de p^*^	IC 95%
Sexo	2,163	4,357	0,037	1,048 - 4,465
Segurança - vacina (C4)	0,317	8,712	0,003	0,148 - 0,680
Constante	0,896	0,099	0,753	

* *teste qui-quadrado; IC - Intervalo de confiança; variável de resposta: hesitação vacinal. Categorias de referência: sexo (mulher), segurança vacina (não); tabela de classificação: 72,2%.*

## DISCUSSÃO

O rápido desenvolvimento das vacinas contra a COVID-19 e a urgência de sua aplicação para conter a pandemia geraram questionamentos sobre segurança e eficácia, contribuindo para a baixa adesão à vacinação^(9)^. Apesar dos benefícios comprovados, observou-se que parte dos profissionais de saúde ainda apresenta hesitação em manter o esquema vacinal, comprometendo a proteção individual, o controle da doença e o alcance das metas de cobertura^(27)^. Neste estudo, essa hesitação esteve associada, principalmente, ao sexo e ao nível de confiança na segurança das vacinas, evidenciando a influência de fatores sociodemográficos e perceptivos na decisão de continuidade da imunização.

Entre as variáveis analisadas, o sexo masculino apresentou maior probabilidade de hesitação em relação à continuidade do esquema vacinal contra a COVID-19, quando comparado ao feminino. Achados semelhantes foram descritos no Brasil, onde estudo com 173.178 participantes identificou associação significativa entre sexo masculino e hesitação vacinal (OR=1,62)^(28)^, e na Venezuela, onde homens tiveram maior proporção de não vacinados em relação aos vacinados (p=0,037)^(29)^. Pesquisas na Polônia apontam que mulheres demonstraram maior disposição para receber doses de reforço atribuída à percepção mais elevada da importância das medidas preventivas^(30)^ e à preocupação com as consequências da COVID-19^(31)^. Em contrapartida, homens mostraram menor engajamento preventivo, influenciados por atitudes de masculinidade e sensação de invulnerabilidade^(32,33)^, o que pode ter contribuído para maiores taxas de infeção entre homens durante a pandemia^(34)^.

Apesar disso, nem todos os contextos apresentam o mesmo padrão. Estudos realizados na Tailândia^(35)^ e na Áustria^(36)^ observaram menor adesão entre mulheres, atribuída à crença de que a vacinação poderia causar infertilidade^(37)^. Além disso, pesquisa com profissionais de saúde em 23 países não encontrou associação entre sexo e hesitação vacinal^(38)^, mostrando que esse fator pode variar segundo aspectos culturais, sociais e informacionais.

A hesitação vacinal entre profissionais de saúde é especialmente preocupante, pois, além de comprometer a cobertura entre esses trabalhadores, influencia diretamente a aceitação da população, dado que um profissional hesitante tende a não recomendar ativamente a vacinação^(36,39)^. Esse comportamento aumenta o risco de infecção tanto para os próprios profissionais quanto para os pacientes, visto que muitos casos graves e óbitos ocorrem em não vacinados^(40)^. A continuidade do esquema vacinal com doses de reforço é necessária para manter altos níveis de imunidade, como demonstrado por estudo que avaliou a imunogenicidade da AstraZeneca após intervalos prolongados entre doses^(41)^.

A necessidade de reforços é reforçada pela alta exposição ocupacional dos profissionais de saúde e pelo surgimento constante de variantes, que contribuem para o declínio da imunidade e redução da eficácia vacinal^(42)^. Revisões indicam queda significativa da proteção cerca de seis meses após o esquema primário^(42)^, justificando a recomendação de reforços anuais, especialmente nesse grupo de risco^(43)^. Ensaios clínicos, como o de avaliação da BNT162b2 (*Pfizer-BioNTech*), confirmam aumento significativo da resposta imune após a dose adicional^(44)^.

Ainda assim, mesmo diante de evidências robustas sobre o benefício dos reforço^(45)^, parte dos profissionais mantém hesitação, com destaque para maior atraso entre enfermeiros em relação a outras categorias^(46)^. Entre os determinantes clássicos da hesitação (confiança, conveniência e complacência), a confiança na segurança da vacina foi um dos preditores mais relevantes neste estudo, corroborando achados da Índia^(47,48)^ e de pesquisa multinacional com profissionais de 22 países^(49)^. A falta de confiança pode decorrer de dúvidas sobre eficácia, receio de eventos adversos e descrédito nas autoridades ou indústrias farmacêuticas^(36,50)^.

Embora não tenha havido associação estatisticamente significativa, mais da metade dos participantes relatou ESAVI. Pesquisas em diferentes países sugerem que o medo de ESAVI é um fator que potencializa a hesitação, como observado em estudos na Índia^(47)^, na Ásia e África^(50)^, e em outras investigações internacionais^(48,51)^.

### Limitações do estudo

Uma limitação deste estudo está relacionada ao seu delineamento transversal, que não permite estabelecer relações de causalidade entre os fatores analisados e a hesitação vacinal, possibilitando apenas a identificação de associações. Soma-se a isso o uso de questionário autoaplicado, que pode ter introduzido viés de informação, seja por lembrança imprecisa (viés de memória) ou pela tendência dos participantes em fornecer respostas socialmente desejáveis.

### Contribuições para a área da enfermagem, saúde ou políticas públicas

O estudo aborda a hesitação na continuidade da vacinação contra a COVID-19 entre profissionais de saúde, comportamento que ameaça a segurança desses trabalhadores e dos pacientes, reforçando a necessidade de ações que incentivem a adesão ao esquema vacinal completo. Para a prática da enfermagem, os achados indicam a importância de estratégias educativas contínuas que capacitem os profissionais a atuar como multiplicadores de informações seguras e baseadas em evidências. Na saúde coletiva, contribuem para o planejamento de intervenções direcionadas a grupos específicos, considerando fatores sociodemográficos e perceptivos. No âmbito das políticas públicas, ressaltam a necessidade de comunicação clara, transparente e segmentada para combater a desinformação, aumentar a confiança nas vacinas e manter altas coberturas vacinais.

## CONCLUSÕES

A análise dos fatores associados à hesitação na continuidade do esquema vacinal contra a COVID-19 evidenciou que a confiança na segurança das vacinas é determinante para a adesão entre profissionais de saúde, enquanto o sexo também se mostrou um fator relevante na formação de atitudes diante da imunização. Observou-se que parte desses profissionais, especialmente da equipe de enfermagem, apresenta resistência à continuidade da vacinação, o que reforça a necessidade de estratégias direcionadas para este grupo.

Os achados deste estudo podem subsidiar a formulação de intervenções que assegurem a adesão à continuidade do esquema contra COVID-19, com destaque para ações que desmistifiquem crenças e informações equivocadas sobre os imunizantes, promovendo transparência quanto à eficácia e segurança das vacinas, e reforçando seus benefícios no controle de doenças imunopreveníveis.

Adicionalmente, ressalta-se a importância de desenvolver investigações que aprofundem a compreensão dos fatores que influenciam a decisão de vacinação entre profissionais de saúde, possibilitando a implementação de estratégias efetivas nos serviços de saúde capazes de aumentar a aceitação e garantir a proteção desse grupo e da população que assiste.

## Data Availability

Os dados de pesquisa estão disponíveis no corpo do artigo.
